# *Campylobacter* prophage diversity reveals pervasive recombination between prophages from different *Campylobacter* species

**DOI:** 10.1128/spectrum.02795-23

**Published:** 2023-12-13

**Authors:** Adán Manuel Piña-González, Hugo G. Castelán-Sánchez, Juan Manuel Hurtado-Ramírez, Gamaliel López-Leal

**Affiliations:** 1 Laboratorio de Biología Computacional y Virómica Integrativa, Centro de Investigación en Dinámica Celular, Universidad Autónoma del Estado de Morelos, Cuernavaca, Morelos, México; 2 Grupo de Genómica y Dinámica Evolutiva de Microorganismos Emergentes, Consejo Nacional de Humanidades, Ciudad de México, México; 3 Instituto de Biotecnología, Universidad Nacional Autónoma de México, Cuernavaca, Morelos, México; University of Pittsburgh School of Medicine, Pittsburgh, Pennsylvania, USA

**Keywords:** *Campylobacter*, prophages, phages, mobile genetic elements, prophage evolution, bacteriophages, livestock

## Abstract

Prophages, bacteriophages integrated into bacterial genomes, play a critical role in the genetic diversity and evolution of bacterial species. In this study, we investigated the prophage population harbored in *Campylobacter* species. A total of 446 high-quality and complete genomes of *Campylobacter* isolated from different sources revealed 431 prophages. We analyzed the phylogenomic relationships between 490 phages and prophages, unveiling a wide diversity of undescribed prophages. Bacteriophage pangenomes revealed shared genetic repertoires among different phage genera. In addition, we found recombination events between prophages from different phage genera, mainly in prophages infecting *Campylobacter jejuni* and *Campylobacter coli*, showing distinct evolutionary histories in specific regions of the prophage genomes. Specifically, these regions showed variations in gene content, enrichment in structural genes and others in hypothetical proteins, genes involved in transcription, DNA-associated factors, and genes associated with lysis. Finally, *Campylobacter* prophages alter the host genetic repertoire by carrying virulence factors and antibiotic-resistance genes.

**IMPORTANCE:**

Prophages play an important role in shaping the genetic diversity and evolution of their hosts. Acquisition or loss of prophages can lead to genomic variations, including changes in the bacterial phenotype promoted by recombination events, genetic repertoire exchanges and dissemination of virulence factors, and antibiotic resistance. By studying prophages in *Campylobacter* species, scientists can gain insights into the evolutionary patterns, pathogenicity mechanisms, epidemiology, and population dynamics of these species. This has implications for public health, antibiotic resistance surveillance, and the development of targeted therapeutic approaches.

## INTRODUCTION

*Campylobacter* is a significant public health concern because it is the most prevalent foodborne pathogen responsible for gastroenteritis worldwide (1, 2). *Campylobacter* species can colonize a ubiquitous range of environments, from poultry, companion pets, and livestock to humans (3, 4). *Campylobacter jejuni* is the most common species responsible for human infections and is a leading cause of bacterial food poisoning in humans (1, 3, 5). Over the last decade, *Campylobacter* species have become one of the most prominent bacterial pathogens in public health because of the emergence of multidrug-resistant (MDR) strains (6, 7). In addition, it has been observed that several species within the *Campylobacter* genus can exchange genetic material between each other and with other bacterial genera (7 – 9).

The spread of antimicrobial resistance is primarily facilitated by horizontal gene transfer (HGT), mediated by many mobile genetic elements (MGEs) that facilitate the acquisition of antibiotic-resistant genes (ARGs) and virulence factor genes (VFGs). Moreover, this group of bacteria is widely distributed in different environments and organisms, as mentioned previously (10 – 12). Therefore, the interconnection of these factors promotes the exchange of genetic material, resulting in pathogenic phenotypes in both humans and animals. Although plasmids, gene cassettes, and insertion sequences have been relatively well studied in these species (7, 9, 13), other types of MGEs have not yet been as well characterized, bacteriophages being one of them.

Bacteriophages (phages) are considered the most abundant biological entities in the biosphere (14). These phages reproduce either through a lytic cycle, like virulent phages, or vertically by integrating into the host genome and taking advantage of their replication cycle as prophages (15). Both phages and prophages are responsible for modulating bacterial populations by modulating host abundance or by providing them with genes that benefit the host, such as ARGs or VFGs (16 – 18). In the past, identifying prophage regions was a computational challenge due to the limited knowledge on phage sequence diversity. However, in recent years, the development of powerful computational tools, the growth of viral databases, and the combination of different tools have enabled efficient identification of prophage regions (19). As a result, the unveiling and characterization of phage and prophage populations is now possible (20).

*Campylobacter* phages have been studied mainly from epidemiological aspects, where most reported phages mainly infect *C. jejuni* and *Campylobacter coli* (21, 22). In addition, the classification of *Campylobacter* phages has not been fully explored and standardized. For example, lytic *Campylobacter* phages are classified into three groups according to their genome size (22, 23). Group I contained uncharacterized phages with large genomes (320–425 kbs), whereas groups II and III included phages with genomes between 175 and 183 kbs (*Firehammerviruses*) and 131–135 kbs, respectively (*Fletcherviruses*) (21). Additionally, according to the National Center for Biotechnology Information (NCBI) database, there are 11 families and 11 genera of *Campylobacter* phages, of which only 59 phage genomes are complete (accessed in late February 2023). *Fletcherviruses* and *Vequintaviruses* were the most frequently reported. Clearly, there is a lack of knowledge regarding *Campylobacter* bacteriophage diversity, which needs to be addressed to understand the impact of bacteriophages on the *Campylobacter* species evolution. In this study, we explore the diversity of phages and prophages across different species of the *Campylobacter* genus. A total of 490 bacteriophages were analyzed using comparative genomics and phylogenomics to provide a comprehensive characterization of phage diversity.

## MATERIALS AND METHODS

### Genomes used and prophage identification

To conduct our study, 446 complete bacterial genomes of the *Campylobacter* genus were downloaded from NCBI (Supplementary material 1). The quality of the genomes, namely, completeness and contamination percentages, was determined using CheckM (24). Only complete and uncontaminated genomes were included (completeness ≥95% and contamination ≤5%). Biosample information for all genomes was obtained using efetch form E-utilities (25). We collected the metadata for the following sections: isolation source, isolation site, host, environmental medium, and sample type. We then categorized the isolates as Avian (corresponds to non-domesticated birds), Reptile, Environmental, Mammal (non-farm mammals), Insect, Human, Crustaceans, Livestock (includes any farm animal such as Chicken, Turkey, Bovine, Swine, and Sheep). Prophage predictions were carried out using VirSorter2 (26), and the quality of the prophage genomes was checked using CheckV (27). We followed the publicly available protocol for the validation of the first-instance prophage prediction (dx.doi.org/10.17504/protocols.io.bwm5pc86). Briefly, the final quality prophages analyzed by CheckV were validated in a second screening using VirSorter2. All genomes were annotated using prokka (28) and pharokka (29) for bacterial and viral genomes, respectively.

### Homologous groups and phylogenetic reconstruction

We aimed to construct homologous groups from *Campylobacter* bacterial and bacteriophage genomes to gain insights into their evolutionary relationships. For bacterial genomes, we ran roary (30), setting the BLAST search parameters to a length coverage of ≥90% and an amino acid sequence identity of ≥80%. We created homologous groups (HG) with only one copy gene per genome, which we referred to as single-gene families (SGFs). SGFs were considered for the phylogenetic analysis. The SGFs were aligned with MUSCLE, specifying 50 iterations (31). Then, to create a DNA alignment in frame, we used the program TRANALING, which is part of the EMBOSS suite (32). Additionally, we discarded SGFs with recombination signals using PhiPack (33) with a *P*-value cutoff of 0.05. Then, the SGFs that did not show recombination signals were concatenated to form a super-alignment. Based on this alignment, we constructed a maximum likelihood (ML) phylogeny, selecting GTR + F + I + G4 as the best model suggested by IQTree (34). We ran a nonparametric bootstrap analysis (100 replicates) on the ML-phylogeny to establish the support for the clades. To construct homologous groups from the bacteriophage genomes, the protocol described above was modified on the BLAST parameters to a length coverage of ≥70% and an amino acid sequence identity of ≥40% (35). To infer the nucleotide-based intergenomic similarities between phages and prophages, VIRIDIC with default parameters was used. The species and genus levels corresponded to 95% and 70% similarity, respectively (36). The neighbor-joining tree was constructed by ape R-package (37) using the distance-based matrix from VIRIDIC.

### Virulence and antibiotic resistance genes

We employed the Comprehensive Antibiotic Resistance Database (CARD) (38) to identify antibiotic resistance genes (ARGs) and virulence factors (VFGs) (39) in all *Campylobacter* bacterial species and bacteriophage genomes. All hits with ≥80% coverage between the query and the target (subject), and with ≥80% amino acid sequence identity, were considered. Antibiotic drug classes were also determined using the CARD’s Antibiotic Resistance Ontology (ARO) ID. We also collected the identifiers from the virulence factor database (VFDB) (VCF) to assign classes of VFGs.

### Recombination analysis

RDP5.5 (40) was used to assess for recombination using all its methods with default parameters. Prophages with phylogenetic abnormalities detected by six or more methods and with a probability of <10^−10^ for each method were reanalyzed using GARD (41). The confirmed recombinants were then considered as true recombinants. Individual phylogenetic trees were inferred by VICTOR (42) using the splitting regions according to the breakpoint coordinates (using the formula d4 recommended by VICTOR for incomplete viral genomes). Nucleotide diversity was determined using the nuc.div function in the pegas R-package (43).

## RESULTS

### The wide spread of prophages in *Campylobacter* species

**Fig 1:**
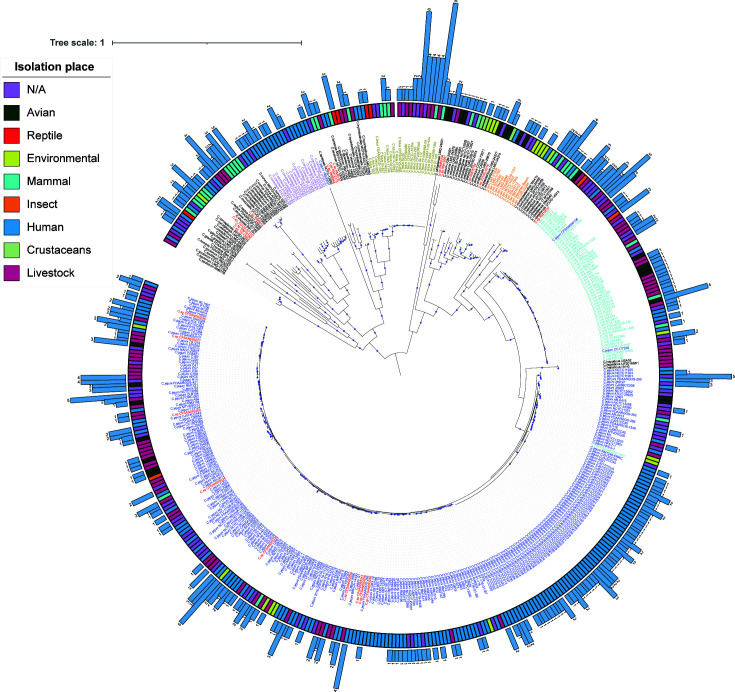
Maximum likelihood (ML) phylogeny shows the relationships among all the isolates. The external circle provides the isolation source of the isolates when available. Bar plots next to the isolation source circle denote the number of prophages identified for each isolate. The colors of the labels denote the most abundant *Campylobacter* species, namely, *Campylobacter jejuni* (blue), *Campylobacter coli* (cyan), *Campylobacter fetus* (brass), *Campylobacter* sp. (red), *Campylobacter concisus* (lavender) and *Campylobacter lari* (orange). The tree scale is the number of substitutions per site, and bootstrap values higher or equal to 90 are depicted with blue circles at the internal nodes of the phylogeny.

To explore the abundance of prophages in *Campylobacter* species, we analyzed only high-quality and complete bacterial genomes (see Materials and Methods). We kept 446 genomes comprising 34 different species (listed in Supplementary material 1). Of these, *C. jejuni* (58%) and *C. coli* (12%) were the most abundant in the data set. In addition, the genomes used were isolated from different sources, such as humans, birds, insects, reptiles, livestock, and environmental samples (Supplementary material 1). We identified a total of 431 prophages with an average of 0.96 per bacterial genome. To obtain a comprehensive picture of the prophage populations in *Campylobacter* species, we first constructed the phylogenetic relationships of the *Campylobacter* species using 71 SGFs (46,749 nucleotides per genome) without recombination signals (see Materials and Methods). We first note a well-supported major clade (blue labels) that corresponds to *C. jejuni* (Fig. 1, blue labels) as well as the clades for *C. coli* (cyan labels) and *Campylobacter fetus* (brass labels). In addition, the ML-phylogeny revealed incomplete classification in some genomes (44, 45). For example, some genomes annotated in the NCBI as *Campylobacter* spp. (red labels) fall within the (well-supported) clades of *C. jejuni* or *C. coli* (Fig. 1), and a few *C. jejuni* or *C. coli* were placed outside their clades.

Nevertheless, the ML-phylogeny of the *Campylobacter* species was consistent with previous studies (46). It is important to note that in some cases, the phylogeny showed a cluster in the *C. jejuni* clade, according to the isolation source. This suggests that a particular population structure is associated with the isolation source. In general, however, we observed that isolates were interspersed with isolates from other sources, suggesting that these isolates may have been a reservoir for spread to other hosts. The ML-phylogeny in the context of phage abundance revealed that 62.27% of the genomes contained at least one prophage. In addition, some *C. fetus* genomes harbored up to nine prophages (Fig. 1). Interestingly, when considering only isolate sources with more than 10 genomes, we noted that the livestock and human isolates showed the highest average number of prophages per genome (1.73 and 1.32, respectively). Furthermore, considering the polylysogens, *C. fetus* CFViADRI1362 and *C. fetus* CFViADRI545 isolated from livestock showed the highest number of prophages in the data set, with nine and eight prophages, respectively. However, no significant difference (Wilcoxon test *<*i>*P*-value > 0.05) was found in the abundance of prophages with respect to the isolation sources. In other words, genomes harboring many prophages are particular events that do not correlate with the source of the isolate.

### The evolutionary relationships of the prophages are related to their hosts

**Fig 2:**
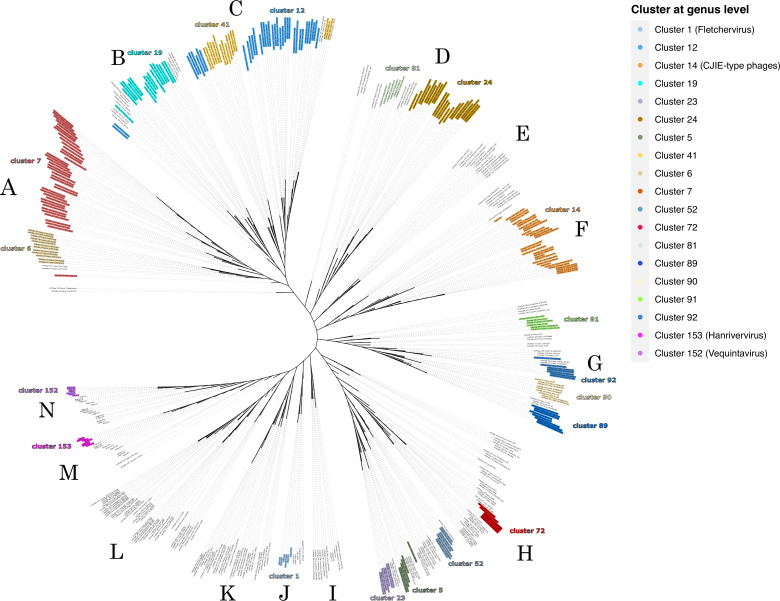
The neighbor-joining (NJ) phylogenetic tree was constructed with 490 phage complete genome sequences using the intergenomic similarity network-related phages calculated by VIRIDIC (see Supplementary material 3). Only genera with more than five members (bacteriophages) were colored. The clusters at the genus level corresponded to 70% similarity (see Materials and Methods). The color labels are found on the outer edge of the tree. The discrete groups observed in NJ-tree (**A–N**) were assigned arbitrarily.

To understand the phylogenetic context of the prophage population, we inferred intergenomic similarities by comparing 431 prophages in the context of other previously reported *Campylobacter* bacteriophages (59 genomes) at the NCBI (Supplementary material 2). In general, prophages showed a strictly narrow genomic host range at the species level (47). In other words, most prophages of the same phage species were found in the same host species (Supplementary material 3). However, we found that phage_400 and phage_398, which belong to the same phage-species, were present in *C. coli* 14158–6 and *C. jejuni* 15065A strains, respectively (Supplementary material 3). We also identified prophages of the same genus in *C. coli* and *C. jejuni* (Supplementary material 3), indicating that some prophages of the same genus or species have a broad host range and can infect different *Campylobacter* species hosts (48, 49). We then constructed a neighbor-joining tree based on the bacteriophage distance matrix (see Materials and Methods). We observed 14 major groups (named group A–N), which were generally grouped according to their host species (Fig. 2). For example, group A is mainly comprised of clusters 6 and 7 at the genus level, corresponding only to *C. jejuni* prophages. The same observation applies to group C, composed of two phage genera (genus-level clusters 12 and 41) identified in *C. jejuni*. Furthermore, group B mainly comprised phages infecting *C. coli* except for prophage_227, prophage_229, prophage_351, and prophage_429, which were found in *C. jejuni* (Fig. 2). In addition, the prophages located in group D corresponded only to the *C. jejuni* prophages. Interestingly, all the prophages of these groups (A, B, C, and D) showed a degree of similarity (with a coverage of 20%–30% and identity, ~97%) with the *Campylobacter jejuni* integrated element (CJIE)-like prophages previously reported as unclassified Caudoviricetes (50). Notably, these groups were mainly represented by seven different genera (Fig. 2) and corresponded to groups of prophages identified directly from the host genome, suggesting that they may be new phage lineages unveiled. Moreover, the prophages of group F corresponded to CJIE-type phages (80%–90% of coverage and 90%–98% of identity). This group includes the CJIE phages previously described only in *C. jejuni* (CJIE4-1, CJIE4-2, CJIE4-3, CJIE4-5, and PC10). However, we identified two CJIE-type phages (prophage_400 and prophage_175) in *C. coli* (50). On the other hand, phages previously reported and characterized (Supplementary material 2) were located in groups J, M, and N. For example, *Firehammervirus* and *Fletchervirus* are located in group J; *Hanrivervirus*, *Dhillonvirus*, *Lambdavirus*, and *Rtpvirus* are in group M; and *Vequintavirus*, *Epseptimavirus*, *Phapecoctavirus*, *Justusliebigvirus*, and *Tequintavirus* are in group N. Finally, prophages from groups G, H, K, E, and L showed no similarity to previously reported bacteriophages.

These results indicate that the genomic relationships of prophages are closely related to their hosts. However, it is important to note that some prophages grouped with prophages from distinct host species (prophages from groups H, K, and L), indicating that phages from different genera may share common genetic repertoires. To gain insights into this point, we collected all phage genera with ≥5 prophages, resulting in 239 bacteriophages. Next, we determined the pangenome of the collected phages to obtain a principal component (principal coordinate analysis [PCoA]) based on the pan-matrix. Only 41.7% (239 dimensions) of the total variation was observed along these two directions (Supplementary material 4). However, the first 10 dimensions showed 84.5% of the variance in our data, suggesting that the remainder of the variation was due to the presence of unique homologous groups for each phage. The first principal (horizontal) axis is spanned by three tight clusters of phage genera. Of these, one was composed of the genera 7, 12, 19, and 41. Interestingly, prophages from these clusters infect *C. jejuni* and *C. coli*. Another cluster comprises prophages from genera 5, 23, 52, and 72, also present in *C. jejuni* and *C. coli*. The last tight cluster corresponds to the prophages from clusters 89, 90, 91, and 92 (Supplementary material 4), which infect only *C. fetus*. Finally, these results indicate that prophages from different genera share genetic repertoires and could be subject to recombination events.

### Pervasive prophage recombination

**Fig 3:**
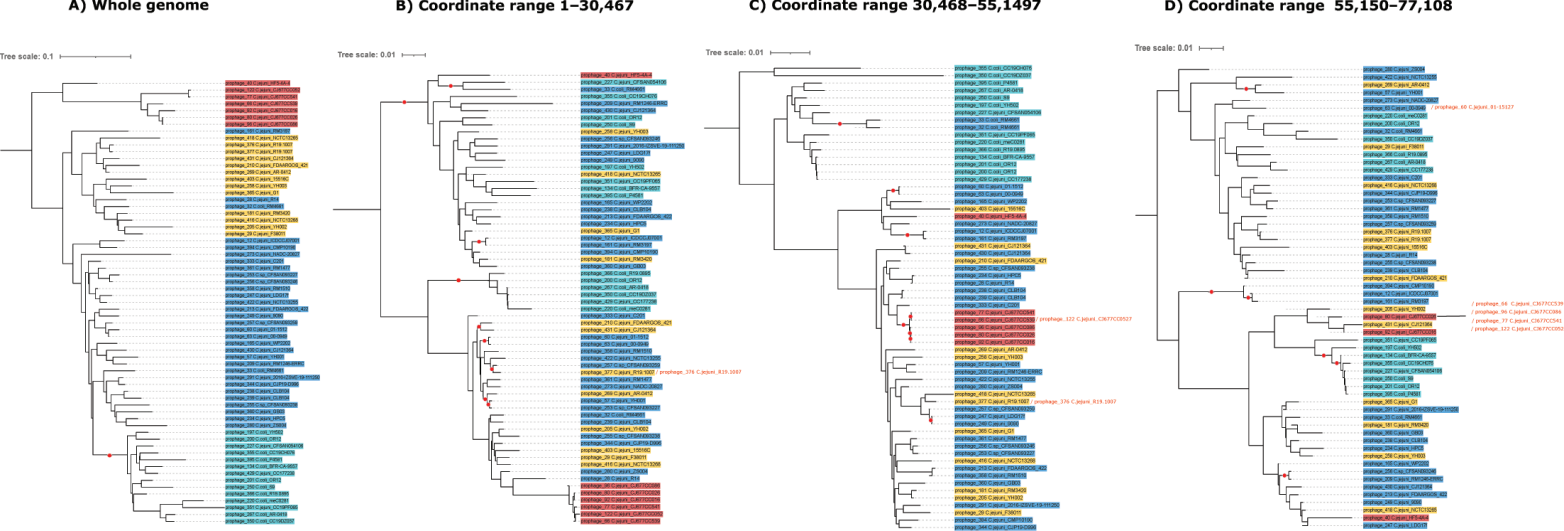
Individual genome BLAST distance phylogenies were reconstructed for the 5′ and 3′ ends of the viral genome (split at breakpoints 30,467 and 55,1497) using nucleotide model d6 for the whole genome (**A**) and d4 for the split phage genome regions (**B–D**). The red dots denote pseudo-bootstrapped support values from 100 replications (only values >50% are shown). The colors of the labels denote phage clusters at the genus level, as shown in Supplementary material 3.

Our observations suggest that recombination probably occurred. To this end, we estimated the putative recombination events between the clusters observed in Supplementary material 4. Our most compelling finding was that GARD identified two putative breakpoints (Supplementary material 5 A) in 65 prophages of different genera (12, 41, 19, and 7), which infect *C. jejuni* and *C. coli* (Fig. 3). Individual phylogenies were then reconstructed for the three regions collected from prophage genomes at breakpoint coordinates Region 1: 1–30,467 bp (Fig. 3B), Region 2: 30,468–55,149 bp (Fig. 3C), and Region 3: 55,150–77,108 bp (Fig. 3D), resulting in independent trees showing distinct evolutionary histories of these regions. Interestingly, several phylogenetic discrepancies were observed between the prophages of genera 12, 41, 19, and 7 (Fig. 3)—clearly, the prophages of genera 19 and 7 clearly form well-defined clades when considering the whole genome (Fig. 3A). However, in phylogenies constructed using genomic segments (Regions 1–3), the prophages of these genera no longer formed discriminatory clades (according to the phage genus) and were scattered throughout the tree (Fig. 3B through D). An important point to note is that the length of the branches in the tree was reduced for the prophages of genus 7 due to the fact that they eventually collapsed into redundant segments (Fig. 3D, red labels). Our data confirm that the nucleotide diversity for Region 1, Region 2, and Region 3 was 0.270, 0.259, and 0.239, respectively. Interestingly, genes coding for structural proteins were enriched in Region 2 (Supplementary material 5B), whereas genes coding for hypothetical proteins, transcription, DNA associated, and lysis were enriched in the segment in Fig. 3B and D (Supplementary material 5B). As in the previous case, we also found a breakpoint in 29 prophages from genera 24 and 81 (Supplementary material 6). However, we found no functional enrichment in the genes encoded in each region (Supplementary material 7A and B). Furthermore, these prophages were found only in *C. jejuni*.

### Prophages altering the host genetic repertory by carrying genes encoding for virulence factor and antibiotic resistance

Previous studies have reported that some *Campylobacter* species can exchange genetic material with each other as well as with other bacterial genera (8). Here, we determined whether prophages could play a potential role in acquiring virulence and antibiotic-resistance genes through transduction events. We then focused on the abundance of ARGs and VFGs encoded in the prophages. We found nine different drug families encoded in 2,055 genes in *Campylobacter* bacterial species. Clearly, Supplementary material 8 shows a vast repertory of drug families, covering a wide range of drug classes (Supplementary material 8). Among these, cephalosporins were the most diverse and abundant, coding for 29 different genes (84.18% of the ARGs found). Interestingly, we found 482 *bla*_OXA-659_ genes encoded in 275 genomes, resulting in an average of 1.7 *bla*_OXA-659_ genes. Although most isolates encoded for one type of antibiotic (53.1% of the total number of genomes analyzed), we found 36 isolates that could be categorized as potentially extensively drug resistant (XDR) because they encoded for more than three different drug families (51) (Supplementary material 9). However, only one prophage had ARGs (Table 1). We found that prophage_381 in *C. coli* FDAARGOS_1466 showed one gene encoded for the tetracycline family (*tet*O). However, different results were obtained for virulence genes. We found 23 virulence genes in nine prophages, where the major contributor was Capsule (Table 1). Furthermore, we observed that most of the prophages (five prophages) with VFGs were retrieved from livestock (all cattle isolates) isolates with an average of 1.8 VFGs per prophage, of which all encoded for the Capsule type of virulence factor. (Table 1). Interestingly, four of these prophages belonged to genus cluster 91 (Supplementary material 10). According to biosample data, the genomes in which these prophages were identified were isolated from different years and geographic regions.

**TABLE 1:** Antibiotic-resistant and virulence factor genes^*a*^

Phage ID	Genus cluster ID	Host	Isolation source	ARG name	Category	Type
phage_381 *C.coli*_FDAARGOS_1466	cluster 128	*Campylobacter coli*	N/D	tet-O (1)	Tetracycline	ARG
phage_53 *C.fetus*_97_608	cluster 91	*Campylobacter fetus*	Livestock (bovine)	Capsule (2)	Immune modulation	VFG
phage_312 C.fetus_CFViADRI545	cluster 91	*Campylobacter fetus*	Livestock (bovine)	Capsule (2)	Immune modulation	VFG
phage_321 *C.fetus*_CFV08A1102-42A	cluster 91	*Campylobacter fetus*	Livestock (bovine)	Capsule (2)	Immune modulation	VFG
phage_324 *C.fetus*_CFV08A948-2A	cluster 91	*Campylobacter fetus*	Livestock (bovine)	Capsule (2)	Immune modulation	VFG
phage_406 *C.fetus*_84–112	cluster 91	*Campylobacter fetus*	N/D	Capsule (1)	Immune modulation	VFG
phage_303 *C.fetus*_CFViADRI1362	cluster 92	*Campylobacter fetus*	Livestock (bovine)	Capsule (1)	Immune modulation	VFG
phage_363 C.*jejuni*_G1	cluster 24	*Campylobacter jejuni*	Human	Flagella (1)	Motility	VFG
phage_370 *C.coli*_R19.0802	cluster 123	*Campylobacter coli*	Human	Capsule (11)	Immune modulation	VFG
phage_424 C*.jejuni*_NCTC11924	cluster 238	*Campylobacter jejuni*	Human	FlpA (1)	Adherence	VFG

^*a*^ The table shows the antibiotic-resistant genes (ARG) and virulence factor genes (VFG) found in the prophage genomes. The values in parentheses denote the number of genes coding for a given category in each prophage.

## DISCUSSION

The use of bacteriophages as an alternative strategy to combat superbugs is becoming increasingly common (52). However, one of the main concerns raised by phage therapy is the bacteriophage-induced host genome evolution (53). Notwithstanding, this idea is still under debate due to the pervasive domestication of prophages, which have lost their ability to reproduce through the lytic cycle. Several studies have shown that prophages are determining factors in the virulence of some pathogens, transferring antibiotic resistance genes, conferring resistance to other bacteriophages, and activating latent prophages (54 – 57). Therefore, it is crucial to explore the diversity of prophage populations to understand the evolution, pathogenicity, and population dynamics of superbugs. Here we analyzed the prophage population retrieved from 446 *Campylobacter* isolates. Of these, 62.27% had at least one prophage, suggesting that lysogens are common in pathogenic bacteria (47). For example, previous studies have estimated that approximately 75% of bacteria are lysogenic and, in some cases, constitute up to 20%–35% of the content of the bacterial genome (47). Although most of the isolates had at least one prophage, livestock isolates tended to be enriched for prophages (Fig. 1; Supplementary material 1). From a public health perspective, it is worth paying attention to the fact that many of the ARGs and VFGs are more frequent in livestock *Campylobacter* isolates (7). In this context, two scenarios could occur. On the one hand, the high prevalence of antibiotic resistance and virulence genes in the livestock isolates could be associated with the constant exchange of these genes mediated by temperate phages (58). On the other hand, the domestication forces on prophages could be more significant in the livestock isolates as most of these are under the constant use of antibiotics (7). For example, we found that ARGs and VFGs encoded in prophages are more common in human and livestock isolates. However, we found that the ARGs were more frequently encoded in the bacterial gene repertory rather than in prophages. We observed that one prophage carried an ARG in their genome, which can potentially spread to other hosts. In contrast, we detected that VFGs were more commonly encoded in prophages. Namely, the capsule was the most frequent VFG category encoded in the prophages. Chromosomally encoded expression of this type of virulence factor is known to be controlled by prophages in *Streptococcus pyogenes*. However, some prophages use the capsule for successful infections, resulting in phage infectiousness according to the capsule serotype. However, the role of the capsule virulence factor encoded in the prophage genome is unclear. Further studies are needed to try to understand the role that prophage-encoded capsule VFGs play in the host range of other phages or in the pathogenicity of their hosts. These results suggest that *Campylobacter* phages could be more commonly involved in the exchange of virulence-associated genes, suggesting that prophages primarily contribute to the acquisition of virulence genes rather than ARGs in *Campylobacter* species, unlike what has been described for other pathogens such as *Acinetobacter baumannii*, *Klebsiella pneumoniae*, and *Pseudomonas aeruginosa* (16 – 18).

In this regard, the combination of ARGs and VFGs encoded in *Campylobacter* prophages is of major concern in human and animal health.

An interesting observation of our results, from the point of view of microbial ecology, is that some phage species and genera could infect different *Campylobacter* host species, particularly between *C. jejuni* and *C. coli*. In this respect, recent studies found that species within the *Campylobacter* genus can exchange genetic material with species of different genera. Although our results show that most prophages have a narrow genomic host range, few *Campylobacter* phages could display a broad host range, implying that these prophages could play an essential role in HGT among different *Campylobacter* species.

One of the most relevant findings of our study is that we uncovered a previously undescribed prophage population that comprised of 318 bacteriophage species and 154 genera. Few studies have described a part of the prophage population only for *C. jejuni* and *C. coli* species (21, 22), resulting in the already-known CJIE-like prophages (50). However, this type of prophage is only a tiny part (Fig. 2, group F) of the population of prophages that *Campylobacter* species harbor. For example, there are five most predominant prophage genera, four of which showed recombination signals (genus_7, genus_12, genus_14, genus_24, and genus_19). In this sense, one of our most relevant results was that we found recombination events between prophages of different genera that could probably infect *C. coli* and *C. jejuni*. Here, three possible scenarios could be speculated. First, the recombination events occur via DNA interchanges between hosts (8). Second, the host genome could be under genomic rearrangements (59). Third, these phages have been exposed to more phages from other populations (60) as foreign temperate or lytic bacteriophages could recombine with the prophages harbored in the host genome (54, 61). Any of the three speculated scenarios could give rise to phage diversification and/or activation of domesticated prophages (54). However, further studies are needed to investigate these scenarios. Overall, this study provides insights into the prophage population in *Campylobacter* species, their evolutionary relationships, recombination events, and their role in shaping the genetic repertoire, particularly in terms of virulence and antibiotic-resistant determinants. Understanding the dynamics of prophages in *Campylobacter* contributes to our knowledge of bacterial evolution, pathogenicity, and potential strategies for controlling *Campylobacter*-related infections.

## SUPPLEMENTAL MATERIAL

Supplementary material 1 spectrum.02795-23-s0001.xlsx List of bacterial genomes used.

Supplementary material 2 spectrum.02795-23-s0002.xlsx List of *Campylobacter* bacteriophage genomes used.

Supplementary material 3 spectrum.02795-23-s0003.xlsx List of phages grouped in clusters at genus and species level.

Supplementary material 4 spectrum.02795-23-s0004.eps Principal component analysis (PCA) was performed on a pan-matrix.

Supplementary material 5 spectrum.02795-23-s0005.eps Recombination breakpoint analysis.

Supplementary material 6 spectrum.02795-23-s0006.eps Individual genome BLAST distance phylogenies.

Supplementary material 7 spectrum.02795-23-s0007.eps Recombination breakpoint analysis.

Supplementary material 8 spectrum.02795-23-s0008.eps ARGs found in *Campylobacter* species.

Supplementary material 9 spectrum.02795-23-s0009.eps Histogram of the ARGs.

Supplementary material 10 spectrum.02795-23-s0010.eps Prophage genomic synteny comparisons.

ASM does not own the copyrights to Supplemental Material that may be linked to, or accessed through, an article. The authors have granted ASM a non-exclusive, world-wide license to publish the Supplemental Material files. Please contact the corresponding author directly for reuse.
